# Unbalance between Excitation and Inhibition in Phenylketonuria, a Genetic Metabolic Disease Associated with Autism

**DOI:** 10.3390/ijms18050941

**Published:** 2017-04-29

**Authors:** Antonella De Jaco, Dalila Mango, Federica De Angelis, Flores Lietta Favaloro, Diego Andolina, Robert Nisticò, Elena Fiori, Marco Colamartino, Tiziana Pascucci

**Affiliations:** 1 Department of Biology and Biotechnologies “Charles Darwin”, Sapienza University of Rome, 00185 Rome, Italy; federica.deangelis@uniroma1.it (F.D.A.); floresl@libero.it (F.L.F.); 2 EBRI-European Brain Research Institute, 00143 Rome, Italy; dalilamango@gmail.com (D.M.); elena.fiori@uniroma1.it (E.F.); 3 Department of Psychology, “Daniel Bovet”, Neurobiology Research Center, Sapienza University of Rome, 00185 Rome, Italy; diego.andolina@uniroma1.it (D.A.); marco.colamartino@uniroma1.it (M.C.); tiziana.pascucci@uniroma1.it (T.P.); 4 Foundation Santa Lucia, IRCCS, 00143 Rome, Italy; 5 Department of Biology, University of Tor Vergata, 00133 Rome, Italy; robert.nistico@uniroma1.it; 6 Cell Biology and Neurobiology Institute, National Research Council, 00143 Rome, Italy

**Keywords:** neurotransmission, excitation and inhibition balance, cognitive delay, prefrontal cortex, neuroligins

## Abstract

Phenylketonuria (PKU) is the most common genetic metabolic disease with a well-documented association with autism spectrum disorders. It is characterized by the deficiency of the phenylalanine hydroxylase activity, causing plasmatic hyperphenylalaninemia and variable neurological and cognitive impairments. Among the potential pathophysiological mechanisms implicated in autism spectrum disorders is the excitation/inhibition (E/I) imbalance which might result from alterations in excitatory/inhibitory synapse development, synaptic transmission and plasticity, downstream signalling pathways, and intrinsic neuronal excitability. Here, we investigated functional and molecular alterations in the prefrontal cortex (pFC) of BTBR-Pah^enu2^ (ENU2) mice, the animal model of PKU. Our data show higher frequency of inhibitory transmissions and significant reduced frequency of excitatory transmissions in the PKU-affected mice in comparison to wild type. Moreover, in the pFC of ENU2 mice, we reported higher levels of the post-synaptic cell-adhesion proteins neuroligin1 and 2. Altogether, our data point toward an imbalance in the E/I neurotransmission favouring inhibition in the pFC of ENU2 mice, along with alterations of the molecular components involved in the organization of cortical synapse. In addition to being the first evidence of E/I imbalance within cortical areas of a mouse model of PKU, our study provides further evidence of E/I imbalance in animal models of pathology associated with autism spectrum disorders.

## 1. Introduction

Several reports suggest an association between autism and inherited metabolic diseases among which phenylketonuria (PKU), suggesting that autism spectrum disorders might represent the end result of a dysfunction caused by a metabolic block in the brain [1]. PKU is the prototypical human Mendelian disease (OMIM 261600; overall incidence of 1 in 10,000) resulting from impaired activity of phenylalanine hydroxylase (PAH), the enzyme necessary to convert phenylalanine (PHE) to tyrosine. This deficiency causes hyperphenylalaninemia (HPA), which is especially harmful for the brain during the first years of life, resulting in variable neurological and mental impairments [2,3,4]. Previous evidence from our group demonstrated that the accumulation of PHE in the brain of BTBR-Pah^enu2^ (ENU2) mice impairs protein levels and enzymatic activity of the tryptophan hydroxylase, the rate-limiting enzyme responsible for serotonin biosynthesis [5], and that the serotonin reduction in the brain causes cortical morphological alterations such as a reduction in the dendritic spine density and maturation [6]. Restoring normal levels of brain serotonin in the ENU2 mice, during the third post-natal week, allowed the recovery of some cognitive functions as well as the morphological maturation of pyramidal neuron dendritic spines in the prefrontal cortex (pFC) [6].

We have investigated functional alterations and molecular rearrangements typically associated with neurodevelopmental disorders in an animal model of PKU in order to explore possible common molecular mechanisms in comorbidity with autism. Alterations in excitatory/inhibitory (E/I) ratio in cortical circuitry have been reported in several animal models for neurodevelopmental disorders in association with cognitive delay [7], providing experimental models to define abnormal molecular mechanisms and to identify new therapeutic targets. Since synaptic transmission is regulated by a plethora of molecules where cell-adhesion molecules are emerging as crucial players [8], we have studied the neuroligin/neurexin (NLGN/NRXN) pathway involved in the maturation of the inhibitory and excitatory synapses [9]. Moreover, genes directly involved in the regulation of the ratio between excitation and inhibition represent risk candidate genes [10]. Copy number variations and/or several single point mutations in the NLGN/NRXN synaptic pathway have been detected in association to neurodevelopmental disorders [11] including autism spectrum disorders [12,13,14,15,16,17].

Here we investigate, for the first time, the functional and molecular features underlying the morphological and biochemical phenotype reported in the pFC of ENU2 mice, the genetic murine model of the most common metabolic inborn error. Our data support the hypothesis that in PKU, unknown mechanisms linked to PHE accumulation lead to a E/I imbalance shifting toward inhibition, accompanied by altered expression levels of specific members of the synaptic family of the Neuroligin proteins, classically linked to autism.

## 2. Results

### 2.1. Analysis of Inhibitory and Excitatory Transmission in Layer II/III of ENU2 pFC

**Figure 1:**
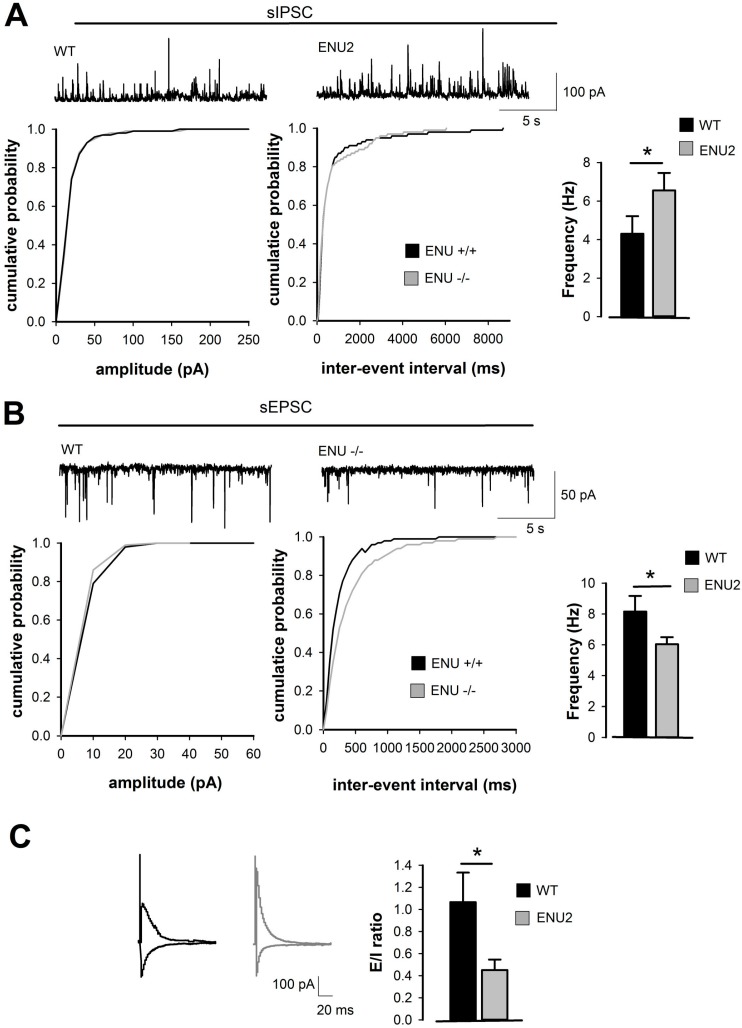
BTBR-Pah^enu2^ (ENU2) mice show altered excitatory/inhibitory (E/I) balance. (**A**) Pooled cumulative distributions of spontaneous inhibitory post synaptic currents (sIPSCs) amplitude (**left**; bin size 10 pA) and inter-event interval (**right**; bin size 50 ms) recorded from neurons of wild type (WT, *n* = 8) and ENU2 (*n* = 7) mice. Representative traces are shown on top. (**B**) Pooled cumulative distributions of spontaneous excitatory post synaptic currents (sEPSCs) amplitude (**left**) and inter-event interval (**right**) recorded from neurons of WT (*n* = 7) and ENU2 (*n* = 6) mice. Histograms are averages (mean ± S.E.M) of the corresponding median values of sEPSCs frequency for the same neurons. Representative traces are shown on top. (**C**) Histograms are averages (mean ± S.E.M) of E/I ratio recorded from WT (*n* = 6) and ENU2 (*n* = 6). Representative traces are shown on the left. (* *p* < 0.05).

ENU2-mutant mice exhibit abnormal behaviors that mimic the intellectual disability symptoms observed in human PKU untreated patients. In order to assess whether immature spine morphology and cognitive impairments described for PKU in the ENU2 mice [6] reflect functionally a different cortical activity in comparison to parental controls, we have measured the spontaneous inhibitory postsynaptic currents (sIPSC) and spontaneous excitatory postsynaptic currents (sEPSC) from layer II/III of brain pFC by using whole-cell patch clamp recordings. We have analyzed synaptic transmission by assessing amplitude and frequency of action potential dependent inhibitory and excitatory spontaneous events from slices obtained by ENU2 and relative control mice at postnatal day 60 (PND 60). We have measured cumulative probability of amplitude and inter-event interval of frequency for sIPSC and sEPSC. As shown in Figure 1a, we have found higher frequency of sIPSC in ENU2 mice compared to wild-type (WT) (K–S test *p* < 0.001, *t*-test *p* = 0.0412 ENU2 *n* = 8 vs. WT *n* = 7, Figure 1A) and significant reduced frequency of sEPSC in ENU2 mice compared to WT (K–S test *p* = 0.0091, *t*-test *p* = 0.0433 ENU2 *n* = 7 vs. WT *n* = 6, Figure 1B). Consistently, the E/I ratio was also significantly reduced in ENU2 mice compared to WT (*t*-test *p* = 0.0306, ENU2 *n* = 6 vs. WT *n* = 6 Figure 1C).

The reported electrophysiological alterations resemble those typically associated with other neurodevelopmental disorders [7], such as autism, where the excitatory and inhibitory balance is functionally impaired and might account for the cognitive phenotype.

### 2.2. Protein Levels of Synaptic Cell Adhesion Molecules in the pFC of ENU2 Mice

Synaptic cell adhesion molecules operate in concert with neurotransmitter receptors to ensure proper function of synaptic circuits [18]. The NLGN/NRXN pathway is currently one of the most studied trans-synaptic codes acting in the organization of the excitatory and inhibitory synapses. The NLGN family is made of four members (1, 2, 3, 4), encoded by different genes, with NLGN1 being specifically localized to excitatory postsynaptic densities while NLGN2 is found in inhibitory postsynaptic specializations and NLGN3 is present at both [19]. NLGNs play a crucial role in the recruitment of neurotransmitter receptors at the synapse and in the control of the E/I balance in the brain [20].

**Figure 2:**
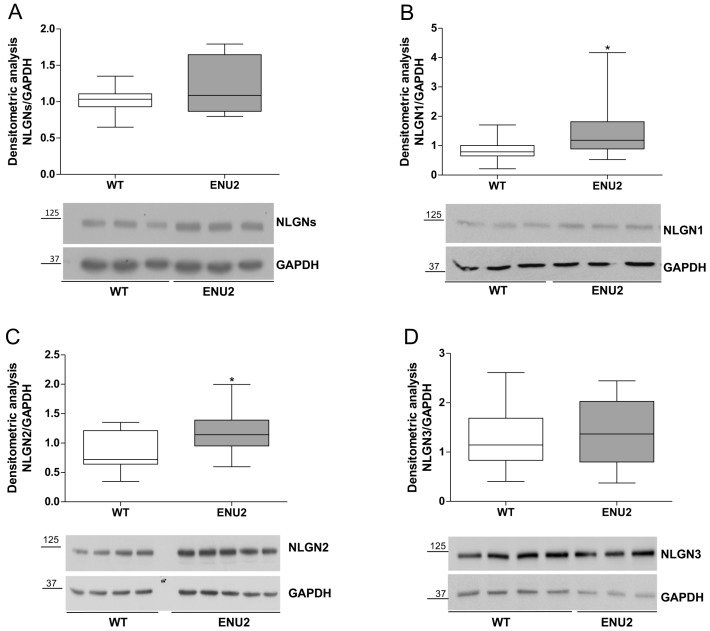
Neuroligins (NLGNs) levels in the pFC of ENU2 mice. Protein levels were quantified by densitometry after western blot analysis for total NLGNs and for NLGN1, 2, and 3 family members. Values were normalized to GAPDH loading control and are represented as a box plot of their distribution (min/max *e* median). (**A**) NLGNs (WT *n* = 7, ENU2 *n* = 8, *p* = 0.2162); (**B**) NLGN1 (WT *n* = 18, ENU2 *n* = 13, *p* = 0.0142); (**C**) NLGN2 (WT *n* = 13, ENU2 *n* = 14, *p* = 0.0266); and (**D**) NLGN3 (WT *n* = 17, ENU2 *n* = 14, *p* = 0.5430). Statistical analysis compared ENU2 values versus WT (* *p* < 0.05). Representative images of western blot analysis are shown. Molecular masses are indicated on the blots in kDa.

We have initially quantified the levels of all the NLGNs by western blot using a PAN-antibody. Analysis of NLGNs levels revealed an increasing trend in the ENU2 mice that however did not present significant results in comparison to WT mice (*t*-test *p* = 0.2162, ENU2 *n* = 8 vs. WT *n* = 7 Figure 2A). We have then investigated the levels of each family member by using antibodies specific for each of the NLGNs forms. We found that NLGN1 (*t*-test *p* = 0.0142, ENU2 *n* = 14 vs. WT *n* = 18 Figure 2B) and NLGN2 (*t*-test *p* = 0.0266, ENU2 *n* = 14 vs. WT *n* = 13, Figure 2C) resulted in increased ENU2 in comparison to WT mice. Non-significant differences were observed for NLGN3 between ENU2 and WT mice (*t*-test *p* = 0.543, ENU2 *n* = 14 vs. WT *n* = 17 Figure 2D). These observations suggest that the functional differences in the ENU2 pFC reflect a different regulation of molecular synaptic components.

## 3. Discussion

Dysregulation of the excitation/inhibition equilibrium has been postulated to represent a hallmark of neuropsychiatric disorders, including autism and some forms of mental retardation. Several mouse models reproducing behavioral phenotypes common to neurodevelopmental syndromes show alterations of the E/I balance. In particular, a mouse model of Rett syndrome showed a shift favoring inhibition in the pFC [21]. Increased inhibition was observed in the somatosensory cortex of mice expressing the R451C autism-related mutation in NLGN3, and this was associated with impairments in social interaction [22]. In general, while a decrease in inhibition is currently associated with autism spectrum disorders, an excess of inhibition has been described to occur in mental retardation syndromes such as Down [23,24,25] and Rett syndromes [21]. Interestingly, by using an optogenetic approach to study real-time effects of elevation of cellular E/I balance in vivo, it was shown that elevated E/I balance resulted in impairments on social behavior that are specific for pFC [26].

Although compelling evidence points toward a link between dysregulation of E/I ratio and behavioral phenotypes resembling those observed in neuropsychiatric disorders, the molecular machinery involved in the regulation of this balance remains unclear.

PKU mice, created by chemically induced genetic mutation, display a phenotype that closely resembles untreated human PKU, characterized by reduced PAH activity, PHE plasma levels 10–20 times greater than those of healthy littermates, impaired cerebral protein synthesis, neurochemical reductions in different brain regions, particularly in serotonergic metabolism in prefrontal cortical areas, reduced functional and morphological synaptic plasticity, and cognitive and other behavioral abnormalities [27].

We have postulated that cognitive impairments might be linked to E/I imbalance and found that there was a resultantly higher inhibition and reduced excitation in the layer II/III of ENU2 pFC, suggesting an overall reduced activity in cortical circuits as observed in other animal models of neurodevelopmental disorders where a shift in the balance between excitation and inhibition—favoring inhibition—has been reported [7].

In recent years, several lines of evidence suggest a possible link between the levels of neuroligins and neurotransmission dysfunctions in association to autism spectrum disorders [28]. Gain and loss of function studies in vitro and in vivo have provided experimental support to the hypothesis that the regulation of the levels of the neuroligin proteins might correlate with alteration of the E/I balance. Transgenic mice where NLGN2 expression has been enhanced showed higher frequency of mIPSC in the pFC and an overall reduction in the E/I ratio [29]. NLGN2 function in modulating inhibitory synaptic currents was further highlighted by the selective deletion of NLGN2 in the medial pFC in a conditional knock-out mouse strain. This resulted in chronic changes in E/I balance characterized by a reduction in frequency and amplitude of inhibitory sIPSCs and by cognitive behavioral changes [30]. This has led us to investigate whether the E/I unbalance in the pFC of ENU2 mice might correlate with altered levels of the NLGNs family members. Indeed, in ENU2 mice we have found different levels of neuroligin proteins in comparison to the parental healthy mice. In particular, our data show unchanged levels for NLGN3 along with higher protein levels for NLGN2 and NLGN1. Enhanced NLGN2 protein levels in the pFC of ENU2 mice correlate with the increased inhibitory transmission observed in the layer II/III and strengthen the hypothesis of a shift in the E/I balance favoring inhibition in the ENU2 mice. In fact, NLGN2 is found in inhibitory postsynaptic specializations [31] where it plays a specific role in the regulation of inhibitory synaptic terminals and in the maintenance of E/I balance in the brain [32]. NLGN2 interacts with collybistin and gephyrin in order to recruit and anchoring GABAA receptors to the post-synaptic membrane [33], favoring the maturation of the inhibitory synapses [34]. At this stage, we have not investigated whether the enhancement of sIPSC is due to an increase of the number of inhibitory synapses. However, the increased levels of NLGN2 cannot explain the decrease in the excitatory neurotransmission in ENU2 mice. Recently, it has been shown that selectively deleting NLGN2 from the II/III layer of pFC leads to a decrease in spontaneous mIPSC without affecting mEPSC [30]. 

The prominent deficit of serotonin in pFC of PKU mice is well documented [5,35] and also a crucial role is played by serotonin in regulating maturational events such as spine morphology through the activation of the serotonin 2A receptor (5HT2A) receptor, expressed in excitatory synapses [36]. Our previous work showed that cortical spine maturation, and consequently cognitive deficits, are affected in ENU2 mice through a serotonin-dependent pathway [6]. Thus, the reduced serotonin release in ENU2 [5] might result in a lower excitatory activity 5HT2A-dependent. This would agree with the data we show in regard to the lower rates of spontaneous EPSCs in the II/III layers of pFC. Our data show a statistically significant increase for NLGN1 in ENU2 mice, classically localized to excitatory synapses [34]. This increase does not agree with the reduced excitatory transmissions found in the layer II/III of the pFC in ENU2 mice. This might be due to the western blot analysis being performed from punchings of the pFC, comprising all of the cortical layers, in contrast to the electrophysiological recordings restricted to layer II/III.

The shift in the ratio from excitation to inhibition is however differentially regulated by the association of the NLGNs with elements critical for synapse formation such as postsynaptic scaffolding proteins, PSD-95 (at the excitatory synapses), and gephryn (at the inhibitory synapses) and to the presynaptic proteins NRXNs [37,38,39]. Therefore, a further analysis of these components will help clarify the mechanism.

The data presented shows the first evidence of E/I cortical imbalance in a genetic murine model of inherited metabolic disease, PKU. The unbalance toward inhibitory transmission in the pFC of ENU2 mice might impact on the proper development of brain circuits involved in cognitive function. The cascade of events that lead from high blood PHE levels to the E/I cortical imbalance in PKU however is still not understood.

Finally, investigating the molecular and physiological mechanisms underlying cognitive disability in PKU mice can provide insights for autism spectrum disorders, as well as for all syndromes characterized by similar pathogenic mechanisms.

## 4. Materials and Methods

### 4.1. Animal Protocols and Housing

All experiments were approved by the ethics committee of the Italian Ministry of Health and conducted under license/approval ID #: 10/2011-B, according with Italian regulations on the use of animals for research (legislation DL 116/92) and the Council Directive 2010/63EU of the European Parliament and the Council of 22 September 2010 on the protection of animals used for scientific purposes. Homozygote (−/−) Pah^Enu2^ (ENU2) and Homozygote (+/+) Pah^Enu2^ (WT) BTBR mice were issued from heterozygous mating. Genetic characterization was performed on DNA prepared from tail tissue using the Easy DNA Kit (Invitrogen, Carlsbad, CA, USA). The ethylnitrosourea (ENU2) mutation was detected after PCR amplification of exon 7 of the Pah gene and digestion with BsmAI restriction enzyme (NEB, USA) as previously described [40]. At PND28, animals (sex matched) were housed 2–4 per standard breeding cage with food and water ad libitum on a 12:12 h dark: light cycle (light on 07.00 a.m.–07.00 p.m. h).

Brain tissue was collected at PND80 from male ENU2 and WT mice. All animals were killed and the brain was removed and stored depending on the experimental procedures. Every effort was made to alleviate animal discomfort and cervical dislocation was applied as the appropriate method of sacrifice.

### 4.2. Slice Preparation for Electrophysiological Recordings

The brain was rapidly removed from the skull and coronal slices (250 μm thick) were cut with a vibratome (VT 1200S, Leica) in cold (0 °C) artificial cerebrospinal fluid (aCSF) containing (in mM): NaCl 124; KCl 3; MgSO_4_ 1; CaCl_2_ 2; NaH_2_PO_4_ 1.25; NaHCO_3_ 26; glucose 10; saturated with 95% O_2_, 5% CO_2_ (pH 7.4), and left to recover for 1 h in aCSF at room temperature.

### 4.3. Whole-Cell Patch Clamp Recordings

Individual slices were placed in a recording chamber, on the stage of an upright microscope (Zeiss, Munich, Germany) and submerged in a continuously flowing (3 mL/min) solution at 30°C (±2 °C). Individual neurons were visualized through a 40× water-immersion objective (Olympus, Tokyo, Japan) connected to infrared video microscopy (Hamamatsu, Hamamatsu City, Japan). Borosilicate glass electrodes (5–7 MΩ), pulled with a PP 83 Narishige puller, were filled with a solution containing the following (in mM): CsCH_3_SO_3_ 115; CsCl 10; KCl 10; CaCl_2_ 0.45; EGTA 1; Hepes 10; QX-314 5; Na_3_-GTP 0.3; Mg-ATP 4.0; pH adjusted to 7.3 with CsOH.

Whole cell patch-clamp recordings have been performed from layer II/III pyramidal neurons of pFC brain slice of WT and ENU2 mice. To isolate sEPSCs and sIPSCs from the same neurons we recorded in voltage clamp mode while maintaining the membrane potential either at the reversal potential for GABA receptor for EPSCs (−70 mV) or at the reversal potential for ionotropic glutamate receptors for IPSCs (+10 mV). To record evoked responses elicited by monopolar stimulating electrodes placed in layer I of pFC, EPSCs and IPSCs were monitored sequentially in the presence of 50 μm APV at postsynaptic holding voltages of −60 and 0 mV, respectively. The E/I ratio is computed as the ratio of excitatory to inhibitory charges, obtained by the integration of the measured currents from the network response triggered by extracellular stimulations [41]. Kolmogorov–Smirnov test (K–S test) and unpaired Student’s *t*-test (*t*-test) have been applied as statistical test with α value set at 0.05, *n* reflected the number of neurons recorded.

### 4.4. SDS PAGE and Western Blot

For protein analysis, frozen brains were removed and dissected to obtain punches of the pFC from brain slices (coronal sections) not thicker than 300 μm. Stainless steel tubes of 1.0 mm inside diameter were used and the coordinates were measured as previously reported [42]. 

Samples derived from pFC punching were homogenized by sonication using RIPA buffer (Life Technologies, Monza, Italy) to extract total proteins and total protein concentration was determined by Bradford assay (Biorad, Rome, Italy). Around 70 micrograms of total proteins were loaded for each sample. Immunoblotting used previously optimized standard techniques [43] including 10% *w*/*v* SDS-PAGE (Biorad, Rome, Italy) and immobilon transfer membranes (Millipore, Bedford, MA, USA). 

Detection of NLGN proteins employed commercial primary antibodies from Synaptic Systems, used at the 1:1000 dilution: anti-NLGN pan mouse monoclonal antibody (clone 4F9, Cat. No. 129-011); anti-NLGN1 mouse monoclonal (Cat. No. 129-111); anti-NLGN2 polyclonal rabbit (Cat. No. 129-203); anti-NLGN3 polyclonal rabbit (Cat. No. 129-113). The anti-GAPDH polyclonal rabbit antibody (abcam ab37168) was used as a loading control. The anti-mouse-HRP and anti-rabbit-HRP (Sigma-Aldrich, Milan, Italy) secondary antibodies were diluted 1:10,000. The HRP signal was developed using the LiteAblot PLUS and TURBO extra sensitive chemiluminescent substrates (Euroclone, Milan, Italy) and exposed to autoradiographic films (Santa Cruz Biotechnology, through Aurogene, Rome, Italy) or revealed by using the ChemiDoc™ MP System (Biorad, Rome, Italy). Densitometry was performed using the Image-J software (version 1.43, NIH, Bethesda, MD, USA). Punching samples were derived from 7 to 18 animals per group and unpaired Student’s *t*-test (*t*-test) statistical analysis was used to compare values from ENU2 and WT mice.

## Abbreviations

sEPSC	spontaneous excitatory postsynaptic potential
sIPSC	spontaneous inhibitory postsynaptic potential
NLGNs	neuroligins
HRP	horseradish peroxidase
SDS	sodium dodecyl sulfate
PAGE	polyacrylamide gel electrophoresis
pFC	prefrontal cortex
LTP	long term potentiation
LTD	long term depression
